# Baseline Seroprevalence of SARS-CoV-2 Specific Antibodies in Hot Spot Areas of Great Tunis, up to 3 Months Post Disease Onset in Tunisia

**DOI:** 10.3390/epidemiologia4020020

**Published:** 2023-06-12

**Authors:** Sonia Dhaouadi, Hejer Letaief, Aicha Hechaichi, Mouna Safer, Rym Moussa, Ridha Bouhali, Fethi Letaief, Latifa Abdelkader, Hamida Ben Salah, Nawel El Mili, Mongi Hammami, Khouloud Talmoudi, Yves Souteyrand, Pierre Nabeth, Mohamed Kouni Chahed, Nissaf Bouafif ép Ben Alaya

**Affiliations:** 1 National Observatory of New and Emerging Diseases, Tunis 1002, Tunisia; 2 Faculty of Medicine of Tunis, University Tunis El Manar, Tunis 1006, Tunisia; 3 Regional Directorate of Health, Ministry of Health, Tunis 1002, Tunisia; 4 Regional Directorate of Health, Ministry of Health, Ariana 2080, Tunisia; 5 Regional Directorate of Health, Ministry of Health, Ben Arous 2033, Tunisia; 6 Regional Directorate of Health, Ministry of Health, Manouba 2010, Tunisia; 7 WHO Regional Office for Eastern Mediterranean, Tunis 1003, Tunisia

**Keywords:** SARS-CoV-2, seroprevalence, rapid antibody test, hot spot areas, household, Tunisia

## Abstract

The extent of the SARS-CoV-2 circulation and the COVID-19 epidemic in Tunisia three months after virus circulation was unknown. The aim of this study was to determine the extent of SARS-CoV-2 infection among household contacts of confirmed COVID-19 cases living in Hot spot areas of Great Tunis, Tunisia by estimating the seroprevalence of antibodies anti SARS-CoV-2 and to identify factors associated to seroprevalence at the first stage of the pandemic in order to guide decision making and to constitute a baseline for further longitudinal analysis of protective immunity to SARS-CoV-2. The National Observatory of New and Emerging Diseases (ONMNE), Ministry of Health Tunisia (MoH), with the support of the Office of the World Health Organization Representative in Tunisia and the WHO Regional Office for the Eastern Mediterranean (EMRO)), conducted a household cross-sectional survey on April 2020 in Great Tunis (Tunis, Ariana, Manouba and Ben Arous). The study was based on the WHO seroepidemiological investigation protocol for SARS-CoV-2 infection. SARS-CoV-2 specific antibodies (IgG and IgM) were qualitatively detected using a lateral immunoassay that detect SARS-CoV-2 nucleocapsid protein and administered by the interviewers. The included subjects were confirmed COVID-19 cases and their households contacts resided in hot spot areas (cumulative incidence rate ≥ 10 cases/100,000 inhabitants) of Great Tunis. Results: In total, 1165 subjects were enrolled: 116 confirmed COVID-19 cases (43 active cases and 73 convalescents cases) and 1049 household contacts resided in 291 households. The median age of participants was 39.0 with 31 years’ interquartile range (Min = 8 months; Max = 96 years). The sex ratio (M/F) was 0.98. Twenty-nine per cent of participants resided in Tunis. The global crude seroprevalence among household contacts was 2.5% (26/1049); 95% CI 1.6–3.6%, 4.8%; 95% CI 2.3–8.7% in Ariana governorate and 0.3%; 95% CI 0.01%–1.8% in Manouba governorate. In multivariate analysis, the associated factors independently related to seroprevalence were age ≥25 years (aOR = 5.1; 95% CI 1.2–22.0), history of travel outside Tunisia since January 2020 (aOR = 4.6; 95% CI 1.7–12.9), symptomatic illness in the previous four months (aOR = 3.5; 95% CI 1.4–9.0) and governorate of residence (*p* = 0.02). The low seroprevalence estimated among household contacts in Great Tunis reflect the effect of public health measures early taken (national lockdown, borders closed, remote work), the respect of non-pharmaceutical interventions and the efficacy of COVID-19 contact-tracing and case management at the first stage of the pandemic in Tunisia.

## 1. Introduction

On 11 March 2020 World Health Organization declared the COVID-19 epidemic caused by the emergent virus: Severe Acute Respiratory Syndrome Coronavirus 2 (SARS-CoV-2) as pandemic. This is the first pandemic caused by a coronavirus and the 6th Public Health Emergency of International Concern [1,2].

The severity of the disease ranged from asymptomatic cases, mild symptom, severe cases (Acute Respiratory Distress Syndrome or ARDS), critical cases and death. However asymptomatic and mild cases can be a substantial source of transmission in the community, representing a significant challenge to prevent human to human transmission. In addition, the substantial proportion of asymptomatic or mild cases is not known since not targeted by most testing strategies and the true burden of the virus circulation may be underestimated [3].

By the end of April 2020, more than 2.8 million cases have been confirmed, with around 193,000 deaths worldwide [4].

Despite the effectiveness of non-pharmaceutical measures in reducing the spread of infection: lockdown, physical distancing, hand hygiene, wearing face mask, school closure, and remote work, left of the measures is closely depended on the extent of infection in the population [5]. Therefore, several seroepidemiological studies have been conducted or are ongoing using different testing methods and different protocols [6].

Determining the SARS-CoV-2 prevalence among people with asymptomatic/mild symptoms or who were never tested despite having symptoms can be a way of monitoring the circulation of the virus in general population, thus helping decisions makers to implement strategies to minimize the risk of transmission, especially in the absence of prophylactic or effective treatment.

In Tunisia, the first case was detected on 2 March 2020. As of April 26, more than 950 cases were confirmed, with 40 deaths and a fatality rate around 4% [7]. The epidemic was mainly (epicenter of outbreak) in the: North (Great Tunis) and South (Kebili and Medenine) of the country.

The extent of the SARS-CoV-2 circulation and the COVID-19 epidemic in Tunisia was unknown three months after the virus circulation in Tunisia.

The objective of this study was to determine the extent of SARS-CoV-2 infection among household contacts of confirmed COVID-19 cases living in Hot spot areas of Great Tunis, Tunisia by estimating the seroprevalence of antibodies anti SARS-CoV-2 (both active and past infections exposure to SARS-CoV-2 virus) and to identify factors associated to seroprevalence at the first stage of the pandemic in order to guide decision making and to constitute a baseline for further longitudinal analysis of protective immunity to SARS-CoV-2.

## 2. Methods

### 2.1. Study Design

The National Observatory of New and Emerging Diseases (ONMNE), Ministry of Health Tunisia (MoH), conducted a household cross-sectional survey with the support of World Health Organization (The Office of the WHO Representative in Tunisia and The WHO Regional Office for the Eastern Mediterranean (EMRO)) on April 2020. The study was based on the World health organization seroepidemiological investigation protocol for SARS-CoV-2 infection [8,9].

### 2.2. Study Setting and Population

**Figure 1:**
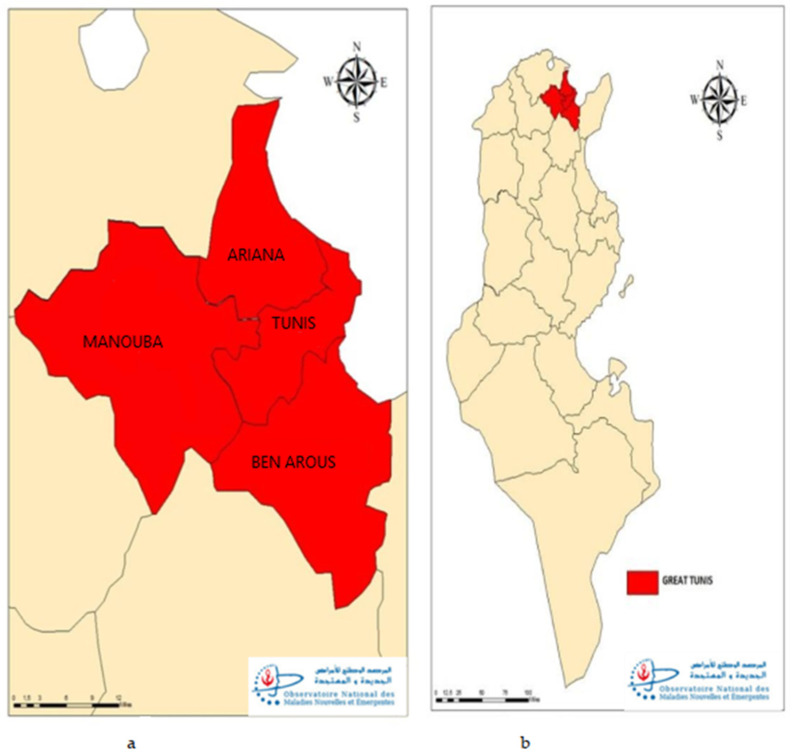
Geographic distribution of participants, seroprevalence survey of SARS-CoV-2, the four.governorates (**a**) of Great Tunis (**b**), Tunisia, April 2020.

This exhaustive study was conducted among all survivors confirmed COVID-19 cases (active and convalescent cases) recorded in our national database (ONMNE) and their household contacts, living in the hot spot areas in Great Tunis is located in North Est of Tunisia and is divided into four governorates: Tunis, Ariana, Manouba and Ben Arous (Figure 1). It accounts 2,863,562 inhabitants in 2020, such 24% of the total Tunisian population.

### 2.3. Eligibility Criteria

#### 2.3.1. Inclusion Criteria

All persons present at the time of the survey in their household, irrespective of their COVID-19 status including children and have given their informed consent, were asked to participate to the study.

#### 2.3.2. Non-Inclusion Criteria

Subjects who refused to give informed consent or to be sampled, or had any contraindication to capillary puncture were not included in this study.

We didn’t include also COVID-19 deaths.

### 2.4. Definitions

#### Case Definition [7]: (According to Our National Guidelines)

- A confirmed COVID-19 case:

Every person, symptomatic or not, meeting the laboratory confirmation diagnostic (Detection of SARS-CoV-2 ribonucleic acid by RT-PCR in a respiratory specimen). The laboratory must be authorized by MoH.

- A close contact was defined as the contact that had experienced any one of the following exposures during the three days before and the 14 days after the onset of symptoms for symptomatic cases or the date on which the sample was taken which led to confirmation for asymptomatic of a probable or confirmed case [7,10]:

- Face-to-face contact with a probable or confirmed case within one meter and for morethan 15 min;
- Direct physical contact with a probable or confirmed case;
- Direct care for a patient with probable or confirmed
- COVID-19 disease without using proper personal protective equipment

- A household was defined as a residential place with a unique address (residential institutions, such as boarding schools, dormitories, hostels or prisons were excluded) [11]
- Index patient is the first confirmed case in a household.
- A household contact: a person (irrespective of age and sex) living in the same household of the index patient during their illness (infectivity period)
- A hot spot area is any area with cumulative incidence rate ≥ 10 cases/100,000 inhabitants.

## 3. Data Collection Tool

### 3.1. a-Questionnaire

A face-to-face interview using a numeric standardized questionnaire was conducted by trained interviewers (health care workers). Five items were recorded: identity of household and participant (age, gender, nationality, number of phone, email address, residence address and occupation); SARS-CoV-2 infection status at the time of the survey (not infected, active or convalescent case), previous exposure in the last four months (travel outside/inside Tunisia, close contact with a suspected or a confirmed case in the previous four months), symptom illness in the previous four months, and Rapid Antibody Test ‘s (RAT) result.

### 3.2. b-Detection of SARS-CoV-2 IgM and IgG Antibodies

SARS-CoV-2 specific antibodies were detected by a SARS-CoV-2 antibody RAT colloidal gold immunochromatography, (Lepu Medical Technology Co.,Ltd, Beijing, P.R.China), a Lateral Flow Immunoassays qualitative IgG/IgM detection system by SARS-CoV-2 nucleocapsid protein (N protein) detection. The test Kit’s performance was internally assessed prior to this survey by a national survey and the sensitivity and specificity were respectively 65.7%; 95% CI 59.7–71.3% and 96.3%; 95% CI 93.0–98.3%. For IgM, sensitivity and specificity were respectively 36.5%; 95% CI 30.8–42.6% and 97.9%; 95% CI 95.2–99.3%. For IgG, sensitivity and specificity were respectively 61.2%; 95% CI 55.2%–67.1% and 97.5%; 95% CI 94.7–99.1% [12]. 

According to manufacturer’s instruction, 20 µL of whole blood sample was added into the sample port followed by adding 2 to 3 drops (80 µL) of dilution buffer. Test kits was visually read after about 15 min; 10–20 min).

We consider a RAT as positive if any band on the test kit indicating the presence of IgM and/or IgG antibodies.

We consider a RAT as negative if none of the IgM and IgG antibodies were detected.

## 4. Data Analysis

Statistical analysis was performed using Epi info software (version 7.2.4 available at https://www.cdc.gov/epiinfo/, accessed on 29 May 2022). The dependent variable (outcome) was seroprevalence (Yes/No) defined as:

- Crude (Apparent) seroprevalence among household contacts (%):

(number of persons tested positive by RAT/total tested household contacts) × 100

- Adjusted (True) seroprevalence for performances of test used (RAT sensitivity and specificity) (%) following this formula [13,14,15];

(1) $$\frac{Crude\;prevalence+Specificity-1}{Sensitivity+Specificity-1}$$

The True prevalence was estimated from apparent prevalence using Bayesian Methods.

- Seropositivity rate among confirmed cases (%):
  (number of confirmed COVID-19 cases tested positive by RAT/total tested COVID-19 cases) × 100

The independent variables included demographic variables (age, gender, governorate of residence), History of travel outside Tunisia since January 2020, Having symptomatic illness in the previous 4 months, Having close contact with a suspected or a confirmed case.

Quantitative variables were expressed by median, interquartile range, minimum and maximum. Qualitative variables were described by number and percentage.

95% Confidence interval (95% CI) of percentage was calculated using exact binomial distribution.

We used ROC (receiver operating characteristic) curve to determine the level of quantitative variable (age) from which the association with seroprevalence was significant.

To establish associated factors to SARS-CoV-2 antibodies seroprevalence we used univariate analysis to establish association between percentages (Chi-square test or Fisher’s exact test when an expected value was less than 5). A *p*-value < 0.05 was considered statistically significant.

Interaction (bivariate analysis) using Woolf Test of Homogeneity. A *p*-value < 0.05 was considered statistically significant.

Independent factors associated to seroprevalence were identified through a multivariate analysis (stepwise: backward logistic regression) using variables significantly associated to seroprevalence in univariate analysis (*p* ≤ 0.2), confounders factors and effect modifier (interaction) were tested. The variables selected in the final model had a *p* < 0.05. Model fitting was tested by Hosmer-Lemeshow test.

## 5. Ethical Considerations

A written informed consent was provided by all participants before participating in this survey. Consent for children under the legal age (<18 years) were obtained from a parent or legal guardian. Each participant was informed that participation in the investigation is voluntary and that s/he is free to withdraw.

Confidentiality of participant was respected: a study identification number by the investigation team for the labelling of questionnaires and specimens was allowed to each participant. All data were analyzed anonymously.

The National Observatory get an official authorization from the national instance of protection of personal data (INPDP) according to any activity related to COVID-19.

A pilot study was conducted in order to evaluate the comprehensiveness of the questionnaire’s items (by interviewers and participants), to identify ambiguous questions, to test the technical problems, to inform about feasibility and acceptability. Relevant modifications were done and the design and the questionnaire were revised and validated for this study.

## 6. Results

In total, 1049 Household contacts of 116 confirmed COVID-19 index cases (73 convalescents and 43 active cases) were enrolled, thus, 1165 subjects, resided in 291 households, were enrolled in the study. The response rate was 88%.

### 6.1. Sociodemographic Characteristics of Participants

**Figure 2:**
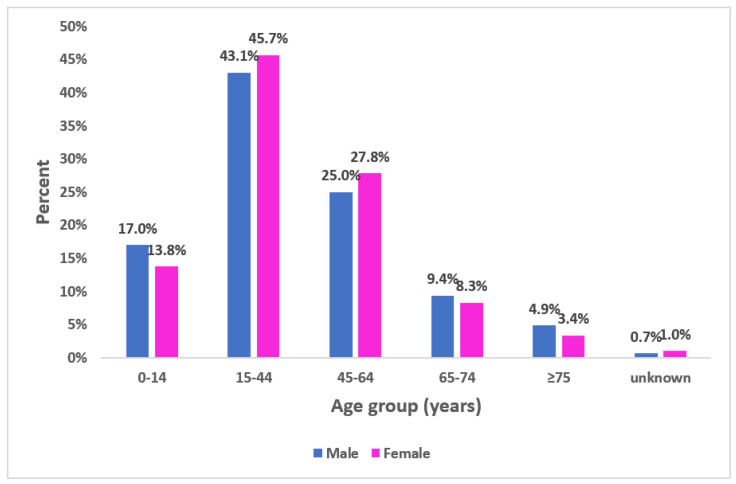
Proportional distribution of participants according to age group and gender, seroprevalence survey of SARS-CoV-2, Great Tunis, Tunisia, April 2020 (*n* = 1165).

The median age of participants was 39.0 with 31 years’ interquartile range (Min = 8 months; Max = 96 years). In total, 517 of participants (44.4%) were aged between 15–44 years (Figure 2). Sex-ratio (M/F) was 0.98.

Three hundred thirty-nine (29.1%; 95% CI 26.5%–31.8%) of surveyed individuals were from Tunis (Table 1). Thirty-one (2.7%) of participants were health care workers.

### 6.2. Symptoms and Exposure History

Among the participants, 196 (16.8%; 95% CI 14.7%–19.1%) had reported symptomatic illness in previous 4 months and 162 (13.9%; 95% CI 12.0%–15.9%) had declared a close contact with a suspected or a confirmed case COVID-19 (Table 2). Among the 105 participant that reported travel history outside Tunisia, the most visited countries were France (38.1%) and Turkey (25.7%).

### 6.3. Seroprevalence of SARS-CoV-2 Infection

In total, 107 RAT were positive for any antibodies (81 for confirmed cases and 26 for household close contacts) thus, the crude global seroprevalence among household contacts was 2.5% (26/1049); 95% CI 1.6–3.6% in Great Tunis.

The seroprevalence by isotype was respectively 1.3%; 95% CI 0.7–2.2% and 1.8%; 95% CI 1.1%–2.8% for IgM and IgG.

The adjusted seroprevalence was 0.8%; 95% CI 0–2.2%.

The seropositivity rate among confirmed cases was 69.8% (81/116); 95% CI 60.6–78.0%: 36.2%; 95% CI 27.5–45.6% for IgM and 64.6%; 95% CI 55.2–73.3% for IgG.

This seropositivity rate was 81.4%; 95% CI 66.6%–91.6% among active cases and 63.0% 95% CI 50.9–74.0% among convalescent cases.

- a-Seroprevalence by governorate of residence:

The seroprevalence was mainly recorded in two governorates: Ariana 4.8%; 95% CI 2.3%–8.7% followed by Ben Arous: 4.1%; 95% CI 2.0%–7.3% (Table 3).

**Figure 3:**
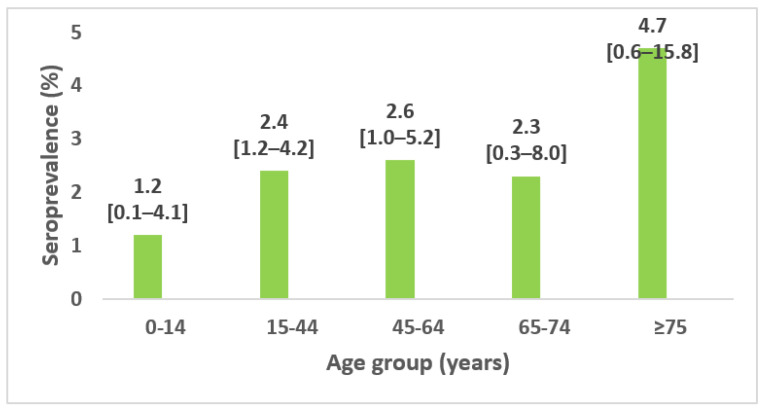
Seroprevalence of SARS-CoV-2 antibodies among household contact by age group and 95% confidence interval, seroprevalence survey of SARS-CoV-2, Great Tunis, Tunisia, April 2020 (*n* = 1049).

- b-Seroprevalence by age:

The seroprevalence increased with age and was the highest among those aged 75 and over: 4.7%; 95% CI 0.6–15.8% (Figure 3).

**Figure 4:**
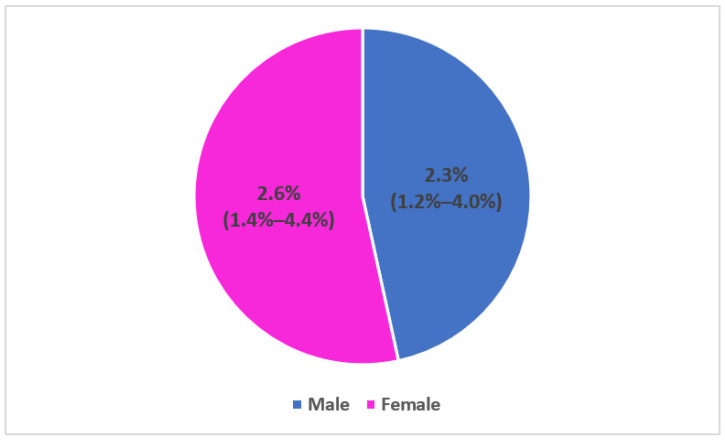
Seroprevalence of SARS-CoV-2 antibodies among household contacts by gender and 95% confidence interval, seroprevalence survey of SARS-CoV-2, Great Tunis, Tunisia, April 2020 (*n* = 1049).

- c-Seroprevalence by gender:

The seroprevalence was not significantly different between gender: Fourteen females among 529 and 12 males among 520 were respectively tested positive for RAT (Figure 4).

- d-Seroprevalence by Date of symptom onset

**Figure 5:**
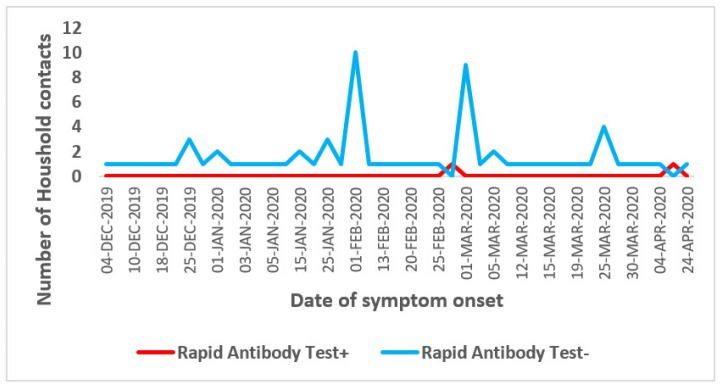
Seroprevalence of SARS-CoV-2 antibodies among household contacts by date of symptom onset, seroprevalence survey of SARS-CoV-2, Great Tunis, Tunisia, April 2020 (*n* = 1049).

Among the 112 (11%) household contacts that reported having symptom compatible with COVID-19 illness in the previous four months, most of them had developed symptom on early February and March 2020 and were tested negative for RAT (Figure 5).

### 6.4. Factors Associated with Seroprevalence Specific Antibodies Anti-SARS-CoV-2

Univariate analysis showed that in Great Tunis, the seroprevalence of specific antibodies was significantly associated to History of travel outside Tunisia since January 2020, age ≥25 years, symptomatic illness in the previous 4 months, close contact with a suspected or a confirmed case and governorate of residence (Table 4). As showed in Table 4, History of travel outside Tunisia since January 2020 had the highest OR, it’s so considered as a principal variable of exposure.

As showed in Table 5, there was no interaction in the association between History of travel outside Tunisia since January 2020 and seroprevalence.

In multivariate analysis, the seroprevalence was independently associated to: age ≥ 25 years (a OR = 5.1; 95% CI (1.2–22.0)), history of travel outside Tunisia since January 2020 (aOR = 4.6; 95% CI (1.7–12.9)), symptomatic illness in the previous 4 months (aOR = 3.5; 95% CI (1.4–9.0)), and governorate of residence (*p* = 0.02) (Table 6).

## 7. Discussion

At the time of this study, 3 months after registered the first case, the Great Tunis, a major administrative and socioeconomic center, and the main source of transmission in the country (half of the cases and half of the deaths at the time of survey), would benefit from being well explored to get an idea of the SARS-CoV-2 circulation.

Our results showed a relatively low seroprevalence of SARS-CoV-2 infection in this region (2.5%). There was substantial geographical variability, with higher prevalence at Ariana (4.8%) and lower at Manouba governorate (0.3%). This is in favor of most of the population appears to have remained unexposed to SARS-CoV-2, even in areas with widespread virus circulation.

In addition, most of participants reported compatible symptom with COVID-19 illness were seronegative. This improve the low circulation of the virus around COVID-19 confirmed cases. To achieve herd immunity, the minimum level of population immunity to fight the spread of infection in the community would be 1-(1/R0) [16].

Having an history of travel outside the country since January 2020 was the mainly factor associated to seroprevalence in univariate analysis. This is explained by the importance of imported case during the first wave of COVID-19 epidemic in Tunisia.

Household contacts aged 25 years and above, history of travel outside Tunisia since January 2020, symptomatic illness in the previous 4 months and governorate of residence were significantly associated to seropositivity in multivariate analysis.

In Ariana, many confirmed cases were self-isolated at home and not in dedicated center that could explained in party the higher seroprevalence observed compared to other governorates.

Those differences in seroprevalence between governorates were also reflected in cumulative incidence rate, which were much higher in Tunis (18.7/100,000 inhabitants) and Ariana (14.4/100,000 habitants) and the lowest in Manouba governorate (9.3/100,000 inhabitants) [7]. In our study, seroprevalence was not significantly varied by gender, children and adolescents were less affected than others. Those facts were also reported by others studies [6,17,18].

RAT for antibodies detection was used for seroprevalence measure: during the study period, ELISA (chemiluminescent microparticle immunoassay) was not yet available. Furthermore, WHO was authorized RAT using for seroprevalence [8].

Comparison between seroprevalence should be done with precautions regarding to the difference in methodologies: study population, study design, study period, type of test used and adjusted for performance’s test.

In the European countries and United Kingdom, seroprevalence ranged between 1.0% in UK (Scotland) to 42.4% in Austria [19]. In addition, different prevalence of antibodies to SARS-CoV-2 were reported by several countries: Spain 5.0%; 95% CI 4.7%–5.4%; Geneva, Switzerland 4·8%; 95% CI 2.4%–8.0% the first week and 10.8%; 95% CI 8.2%–13.9% the fifth week; Wuhan, China 3.8%; Stockholm, Sweden (7.3%), Guilan, Iran 22.0%; 95% CI 19.0%–26.0%; California, USA 4.1%; 95% CI 2.8%–5.6%; Rio de Janeiro, Brazil 4.0%; 95% CI 3.3%–4.7% [6,17,18,20,21,22,23].

### 7.1. Strengths of Study

At the best of our knowledge, this is the first seroprevalence of SARS-CoV-2 infection in Tunisia and among the fewer in the WHO’s EMRO.

Before conducting this study, we internally validated our RDT serological assay through a national survey (sensitivity of 65.7 %; 95% CI (59.7–71.3%) and a specificity of 96.3%; 95% CI (93.0–98.3%)) [12]. After validation of the RDT, we evaluated the seroprevalence in Great Tunis after the first wave of COVID-19 outbreak.

The serological test for the presence of antibodies (IgM or IgG) against SARS-CoV-2 can provide a more accurate estimate of the cumulative prevalence of SARS-CoV-2 infection in a population since the beginning of the outbreak, compared to the molecular test (RT-PCR), in particular IgG resigned for past exposure, are likely to persist for a longer period of time after the viral infection is cleared. Therefore, we can estimate the extent of RT-PCR undetected SARS-CoV-2 infection.

The global cumulative incidence was around 8 cases/100,000 inhabitants as of April 2020. Regarding this low incidence in the Tunisian population, we decided to conduct the sero-epidemiological survey among household contact of confirmed COVID-19 cases in hot spot areas to better estimate the maximum of the seroprevalence.

The study was conducted when lockdown measures were still maintained, which help to maximize the response rate.

### 7.2. Limits of Study

Our study had several limitations. Such seroprevalence studies provide information only about exposure to SARS-CoV-2 and do not provide titer of neutralizing antibodies that would protect recovered patients from secondary infection (reinfection).

Because our study population was not drawn by random sampling, the estimation of the seroprevalence was subject to potential sampling bias and we cannot extrapolate the results to general population, but the study was exhaustive and the results were strength for the study population.

The estimated prevalence reported may be biased (selection bias) due to non-response or that symptomatic persons may have been more likely to participate. This bias is negligible regarding to fewer proportion of symptomatic participants (only 7.1% reported symptomatic illness compatible to COVID-19 in the previous 4 months) and the high percentage of response rate time when our study was conducted.

The sensitivity of the serological test depends on the internal kit’s performance and the test time from the onset of disease. Samples collected from infected individuals outside the time window of antibody response could produce false negatives results, and therefore the observed seroprevalence in our study could potentially underestimate the true prevalence rate of the disease. But adjusting the seroprevalence estimate for performance’s test limit this bias.

Taking into account the cross-sectional design of the study, the dynamic changes of antibody titer in infected individuals over time were not evaluated, inducted a measure bias.

## 8. Conclusions

This study revealed that the prevalence of antibody seropositivity in a highly affected area in Great Tunis varied between 0.3 and 4.8% and was low in global (2.5%). Ariana and Manouba governorates were respectively the most and the less affected areas.

Those finding reflect the effect of public health measures early taken: national lockdown, borders closed, remote work and the respect of non-pharmaceutical interventions and the efficacy of COVID-19 case and contacts management.

These estimates have implications for future public policy guidelines (containment lifting, borders opening) while reinforcing barrier measures (physical distancing, hand washing and face mask wearing).

In light of these results, any proposed approach to achieve temporary herd immunity through natural infection is not only highly unethical, but also unachievable. Starting from the hypothesis that the presence of IgG antibodies is associated with immunity, these results highlight that the epidemic is far from over with a large majority of the population being infection naïve. SARS-CoV-2 virus circulation can quickly and severely return to early outbreak dimensions in a second wave most dangerous than the first once preventive measures are lifted.

## Figures and Tables

**Table 1:** Distribution of participants by governorate of residence, seroprevalence survey of SARS-CoV-2, Great Tunis, Tunisia, Tunisia, April 2020 (*n* = 1165).

Governorate of Residence	Number (%)	95% CI *
Tunis	339 (29.1)	26.5–31.8
Manouba	305 (26.2)	23.7–28.8
Ben Arous	274 (23.5)	21.3–26.1
Ariana	247 (21.2)	18.7–23.7

* CI: confidence interval estimated using exact binomial distribution.

**Table 2:** Characteristics of participants according to symptoms and exposure history, seroprevalence survey of SARS-CoV-2, Great Tunis, Tunisia, April 2020 (*n* = 1165).

Exposures	Number (%)	95% CI *
-Symptomatic illness in the previous 4 months	196 (16.8)	14.7–19.1%
-Close contact with a suspect or confirmed case COVID-19	162 (13.9)	12.0–15.9%
-History of travel outside Tunisia since January 2020	105 (9.0)	7.4–10.6%
-History of travel inside Tunisia since January 2020	54 (4.6)	3.4–5.8%

* CI: confidence interval estimated using exact binomial distribution.

**Table 3:** Seroprevalence of SARS-CoV-2 antibodies among household contact by governorate of residence, seroprevalence survey of SARS-CoV-2, Great Tunis, Tunisia, April 2020 (*n* = 1049).

Governorate of Residence	*n* (%)	95% CI *
**Ariana**	10 (4.8)	2.3–8.7
**Ben Arous**	10 (4.1)	2.0–7.3
**Tunis**	5 (1.7)	0.5–3.9
**Manouba** **Great Tunis**	1 (0.3) **26 (2.5)**	0.01–1.8 **1.6–3.6**

* CI: confidence interval estimated using exact binomial distribution.

**Table 4:** Factors associated in univariate analysis with seroprevalence specific antibodies anti-SARS-CoV-2 among household contacts, seroprevalence survey of SARS-CoV-2, Great Tunis, Tunisia, April 2020 (*n =* 1049).

Exposures	Seroprevalence *n* (%)	Crude Odds Ratio (95% Confidence Interval)	*p*-Value
**-History of travel outside Tunisia since January 2020**			**0.004**
No	20 (2.0)	Ref.	
Yes	6 (9.1)	4.8 (1.9–12.4)	
**-Age group (years)**			**0.03**
<25	2 (0.7)	Ref.	
≥25	24 (3.1)	4.3 (1.0–18.1)	
**-Symptomatic illness in the previous 4 months**			**0.004**
No	18 (1.9)	Ref.	
Yes	8 (7.1)	3.9 (1.7–9.2)	
**-Close contact with a confirmed or a suspected case COVID-19**			**0.01**
No	19 (2.0)	Ref.	
Yes	7 (6.8)	3.6 (1.5–8.7)	
**-Governorate of residence**			**0.02**
Manouba	1 (0.3)	Ref.	
Ariana	10 (4.8%)	**15.0 (1.9–118.5)**	
Ben Arous	10 (4.1%)	12.6 (1.6–99.3)	
Tunis	5 (1.7%)	5.1 (0.6–44.1)	
**-History of travel inside Tunisia since January 2020**			0.2
No	26 (2.6%)	Ref.	
Yes	0 (0%)	-	
**-Gender**			0.7
Female	14 (2.6%)	Ref.	
Male	12 (2.3%)	0.9 (0.4–1.9)	
**-Helath Care Worker**			1
No	26 (2.5%)	Ref.	
Yes	0 (0%)	-	

RAT: Rapid diagnostic test; -: OR cannot be calculated.

**Table 5:** Interaction with History of travel outside Tunisia since January 2020, seroprevalence survey of SARS-CoV-2 among household contacts, Great Tunis, Tunisia, April 2020 (bivariate analysis).

Exposures	Chi-square Woolf Homogeneity Test	Degree of Freedom	*p*-Value
-Age group (years)	0.8	2	0.3
Gender	1.9	1	0.2
-Governorate of residence	0.8	3	0.9
-Close contact with a confirmed or a suspected COVID-19 case	0.009	1	0.9
-Symptomatic illness in the previous 4 months	2.6	1	0.1
-History of travel inside Tunisia since January 2020	*	1	*
-Health Care Worker	*	1	*

*: Chi-square Woolf cannot be calculated.

**Table 6:** Multivariate analysis: Factors independently associated with seroprevalence of SARS-CoV-2 infection among household contacts, seroprevalence survey of SARS-CoV-2, Great Tunis, Tunisia, April 2020.

Exposures	Adjusted Odds Ratio (95% Confidence Interval)	*p*-Value
**-History of travel outside Tunisia since January 2020**		**0.003**
No	Ref.	
Yes	4.6 (1.7–12.9)	
**-Age group (years)**		**0.03**
<25	Ref.	
≥25	5.1 (1.2–22.0)	
**-Symptomatic illness in the previous 4 months**		**0.009**
No	Ref.	
Yes	3.5 (1.4–9.0)	
**-Governorate of residence**		**0.03**
Manouba	Ref.	
Ariana	16.6 (2.1–132.2)	
Ben Arous	11.7 (1.5–93.9)	
Tunis	5.8 (0.7–50.9)	
**-Gender**		0.7
Female	Ref.	
Male	0.9 (0.4–2.0)	

Hosmer test = 0.5.

## Data Availability

Data are available from the corresponding author upon reasonable request.
